# Effects of Short-, Medium-, and Long-Term Treatment Using Photobiomodulation Therapy Combined with Static Magnetic Field in Aging Rats

**DOI:** 10.3390/biomedicines12050990

**Published:** 2024-04-30

**Authors:** Kadma Karênina Damasceno Soares Monteiro, Shaiane Silva Tomazoni, Gianna Móes Albuquerque Pontes, Adeilson Matias Teixeira, Fernanda Aparecida de Araújo Agra, Carolina Barros Alvim, Sâmela Lopes Medeiros Brigato, Rodrigo Labat Marcos, Humberto Dellê, Andrey Jorge Serra, Ernesto Cesar Pinto Leal-Junior

**Affiliations:** 1 Laboratory of Phototherapy and Innovative Technologies in Health (LaPIT), Post-Graduate Program in Rehabilitation Sciences, Nove de Julho University, São Paulo 01504-001, Brazil; 2 ELJ Consultancy, São Paulo 04076-000, Brazil; 3 Post-Graduate Program in Biophotonics, Nove de Julho University, São Paulo 01504-001, Brazil; 4 Post-Graduate Program in Medicine, Nove de Julho University, São Paulo 01504-001, Brazil; 5 Post-Graduate Program in Cardiology, Federal University of São Paulo, São Paulo 04024-002, Brazil

**Keywords:** low-level laser therapy, side effects, adverse events, prophylactic effect, aged rats, toxicity

## Abstract

(1) Background: We investigated the detrimental and protective effects of short-, medium, and long-term treatment with different doses of photobiomodulation therapy combined with static magnetic field (PBMT-sMF) during the aging process. (2) Methods: Rats were treated for 15, 30, and 60 weeks with 1, 3, 10, and 30 J of PBMT-sMF or a placebo control. In addition, eight young rats were not subjected to any procedure or treatment and were euthanized at six weeks old. Skin, muscle, bone, kidney, liver, and blood samples were analyzed. (3) Results: No differences between the groups in the morphology of the skin, muscle, and bone was observed. Glutamic pyruvic transaminase levels were increased in the placebo group after 30 and 60 weeks. Glutamic oxaloacetic transaminase levels were also increased in the placebo group after 30 weeks. An increase in creatinine in the PBMT-sMF 3, 10, and 30 J groups compared with that in the young control group was observed. No significant difference in urea levels between the groups was noted. Vascular endothelial growth factor increased in the PBMT-sMF 10 and 30 J groups after 15 weeks of treatment and in the PBMT-sMF 3 J after 60 weeks. Finally, vascular endothelial growth factor decreased in the PBMT-sMF 30 J group after 30 weeks of treatment. (4) Conclusions: PBMT-sMF did not have detrimental effects on the skin, muscle, bone, kidney, or liver after short-, medium-, and long-term treatments in aging rats. In addition, PBMT-sMF may have protective effects on the muscle tissue in aging rats after short- and long-term treatment.

## 1. Introduction

Photobiomodulation therapy (PBMT) is a nonthermal and non-ionizing light-based therapy that is applied in the form of light amplification by the stimulated emission of radiation (LASER) and light-emitting diodes (LEDs) in the visible and infrared spectra [1]. Irradiated light interacts with the chromophores and photoreceptor cells present in the cytochrome c oxidase (complex IV of the mitochondrial respiratory chain) of different biological tissues. This interaction increases adenosine triphosphate synthesis and, consequently, cell metabolism [1,2,3]. Static magnetic field (sMF) is applied in different fields and its use in biological systems has been increasing in the last years [4]. sMF acts on biological systems through induced electric fields and currents, electron spin, and magneto-mechanical effects [5]. sMF can influence cellular metabolism by acting on ATP (adenosine triphosphate) levels in a time-dependent manner, on biosynthesis, and on stress responses, thus increasing reactive oxygen species production and calcium influx at the cellular level [6,7,8,9]. PBMT and sMF have a similar mechanism of action and they act in a synergistic manner [10]. Therefore, PBMT is used in combination with a static magnetic field (PBMT-sMF) [11,12,13,14,15,16,17] to achieve a greater transfer of electrons [9], thereby increasing the positive effects of both.

Isolated PBMT and PBMT-sMF can decrease pain, modulate inflammatory processes, and regenerate various tissues and conditions [14,15,18]. Specifically, PBMT promotes wound healing [18] and increases collagen production [19], microcirculation, and vascular perfusion in the skin [20]. In addition, PBMT increases oxygen availability [21,22] and redox metabolism [23]. Furthermore, PBMT promotes an increase in the number of myocytes and collagen synthesis in the pathological processes of the skeletal muscle [24,25] in addition to modulating the inflammatory processes and improving muscle regeneration [26,27]. PBMT also improves vascularization and bone tissue quality, increases the amount of new tissue, and decreases healing time, especially following bone injury [28,29]. In addition, studies on animals have provided evidence that PBMT contributes to angiogenesis and the formation of hepatocytes in the hepatic pathological process [30,31]. Although there is little evidence, PBMT is also indicated to be beneficial in preserving renal structure and function [32].

Although the positive effects of PBMT-sMF are well established, evidence regarding its side effects and prophylactic effects remains scarce. A previous animal study showed that treatment with PBMT for two weeks did not cause toxicological abnormalities or changes in tissues such as the bone marrow, liver, kidney, and brain in the long term [33], even when administered trans-cranially [34]. However, the possible toxicity and undesirable effects that may occur due to prolonged treatment with PBMT-sMF remain unclear. In addition, no studies have evaluated the effects of prolonged treatment with PBMT-sMF in intact tissues (with only normal signs of aging) to observe possible prophylactic effects.

Therefore, we aimed to investigate the detrimental and protective effects of short-term (15 weeks), medium-term (30 weeks), and long-term (60 weeks) treatment with different doses (1, 3, 10, and 30 J) of PBMT-sMF during the aging process in rats by analyzing skin, muscle, bone, kidney, liver, and blood samples.

## 2. Materials and Methods

### 2.1. Animals

In total, 128 healthy male Wistar rats weighing approximately 250 g were obtained from the central animal facility of the University of Nove de Julho. The animals were kept under the following standard conditions: temperature (22 to 24 °C), relative humidity (40 to 60%), and a 12 h light/dark cycle. Water and feed were provided ad libitum. All experimental protocols were approved by the Animal Experimentation Ethics Committee of the University of Nove de Julho (Protocol AN0039.2014) and performed in accordance with the standards of the Brazilian College of Animal Experimentation.

### 2.2. Experimental Groups

The animals were randomly divided into six experimental groups as described below:

1. Young-control: eight young animals were not subjected to any procedure or treatment and were euthanized at six weeks old. This group represents the baseline values for all outcome measures
2. Placebo-control: 24 animals were treated with placebo PBMT-sMF.
3. 1 J: 24 animals were treated with PBMT-sMF with dose of 1 J.
4. 3 J: 24 animals were treated with PBMT-sMF with dose of 3 J.
5. 10 J: 24 animals were treated with PBMT-sMF with dose of 10 J.
6. 30 J: 24 animals were treated with PBMT-sMF with dose of 30 J.

All the treatments were initiated at six weeks of age. In each experimental group, 8 animals were treated for 15 consecutive weeks (short-term treatment), 8 animals for 30 consecutive weeks (medium-term treatment), and 8 animals for 60 consecutive weeks (long-term treatment), yielding 24 animals per group.

### 2.3. Treatments

#### 2.3.1. Active PBMT-sMF

A cluster probe with nine diodes (one laser diode of 905 nm, four LED diodes of 875 nm, and four LED diodes of 640 nm, manufactured by Multi Radiance Medical™, Solon, OH, USA) with an adaptor at the tip of the cluster device to reduce the dissipation of the light was used. The optical power of the device was verified every two weeks by a researcher who was not involved in data collection and analysis; for this purpose, a Thorlabs^®^ power meter (Model S322C, Thorlabs^®^, Newton, NJ, USA) was employed. Only one point on the ventral region of the tibialis anterior muscle (bilateral) was irradiated. To irradiate the animals, the spot was kept in direct contact with the skin by applying light pressure to the tissue. Active PBMT-sMF was administered once daily, three times a week (every other day) for 15, 30, and 60 consecutive weeks. A full description of the PBMT-sMF parameters is presented in Table 1. The researcher who administered the treatment was unaware of the allocation of animals to the experimental groups.

#### 2.3.2. Placebo-Control PBMT-sMF

Placebo-control was delivered using the same device as the active PBMT-sMF but without any emission of a therapeutic dose. In placebo mode, the sMF was turned off. The placebo-control group was treated in the same manner as the active PBMT-sMF group.

### 2.4. Collection of Biological Materials

Twenty-four hours after the last therapy session, the animals were anesthetized with a mixture of Ketamine and Xylazine (90 and 10 mg/kg, respectively; König, Avellaneda, Argentina), which was administered intraperitoneally. Subsequently, the skin at the irradiated site, tibialis anterior muscle, and tibial bone were surgically removed bilaterally. The kidneys and liver were surgically excised. Immediately before euthanasia, blood samples were collected for biochemical analysis via a single puncture of the vena cava. The biological samples were processed for further histomorphological and biochemical analyses. After collecting the biological materials, the animals were euthanized with an overdose of a mixture of ketamine and xylazine (300 and 30 mg/kg, respectively).

### 2.5. Outcomes

All analyses were performed by a blinded researcher.

#### 2.5.1. Histological Analysis of Skin, Muscle, and Bone

Histological analysis was performed on the skin, muscle, and bone because these tissues were directly irradiated. Tissue samples from the skin, muscle (longitudinal sections), and bone were collected and stored in 10% buffered formalin for 72 h before histological processing. Subsequently, the samples were dehydrated and subjected to a gradual series of alcohol baths, starting at 50% and progressing to 100% absolute alcohol (LABSYNTH, Diadema, Brazil). The muscles were then diaphonized with Xylol for 4 h (LABSYNTH, Diadema, Brazil) for impregnation and inclusion of the samples in Paraplast^®^ (Sigma-Aldrich, St. Louis, MO, USA). Subsequently, the samples were placed in suitable aluminum containers with molten Paraplast^®^ (Sigma-Aldrich, St. Louis, MO, USA) for 4 h. After impregnation, the samples were placed in a small container covered with molten paraffin wax and left to cure to form a block containing the tissue. For the microtomy, 5 μm microtome (LEICA RM 2125 RT, Leica Biosystems, São Paulo, Brazil) sections were washed and placed in a water bath. Once the samples were prepared, the sections were placed on slides and stained with hematoxylin–eosin dye (muscle, bone, and skin). After staining, the sections were mounted on permanent slides for further analysis under an optical microscope. Slides were photographed using a microphotography system (Olympus System Microscope Model CX 36-Olympus PM10SP Automatic Photomicrographic System). Images of all the groups were obtained using 100× magnification. The images are presented in a similar photographic pattern.

#### 2.5.2. Biochemical Analysis—Glutamic Pyruvic Transaminase (GPT), Glutamic Oxaloacetic Transaminase (GOT), Creatinine, and Urea Levels

GPT, GOT, creatinine, and urea levels were analyzed using spectrophotometry and specific reagent kits (Wiener Lab, Rosario, Argentina). The samples were placed on a plate and the reagent was added. After 90 s, a reading on the spectrophotometer was obtained. The initial absorbance was recorded 1, 2, and 3 min after the first reading. Following this, the mean difference in absorbance/min (∆A/min) was determined by subtracting each reading from the previous one and averaging the values. This average was used for the statistical analysis.

#### 2.5.3. Analysis of Vascular Endothelial Growth Factor (VEGF) by Enzyme-Linked Immunosorbent Assay (ELISA)

VEGF levels in the muscle samples were determined by ELISA using a commercial kit and by following the manufacturer’s instructions (Wiener Lab, Rosario, Argentina). For dosage, the plates were sensitized with a monoclonal antibody referring to the marker of interest and incubated for 12 h at 4 °C. Blocking was performed at the next stage, in which the plates were washed with phosphate-buffered saline (PBS) four times and filled with blocking solution. The plates were incubated again for 2 h at 36 °C and subjected to a new process with four wash cycles. Following this, the samples already diluted previously were added to the plates and remained at 4 °C for another 12 h. After a new wash, streptavidin–peroxidase was added, and the plates were incubated for another hour at room temperature (18–25 °C). The plates were then washed again. Finally, they were revealed, and readings were obtained using a spectrophotometer.

### 2.6. Statistical Analysis

The data were tabulated, and a normal distribution was determined using the Kolmogorov–Smirnov test. One-way ANOVA followed by the Bonferroni post hoc test was performed to verify statistical significance among the groups. Data analysis was performed using the mean values and standard deviation (SD). The level of statistical significance was set at *p* < 0.05.

## 3. Results

### 3.1. Histological Analysis

#### 3.1.1. Short-Term Treatment (15 Weeks)

**Figure 1:**
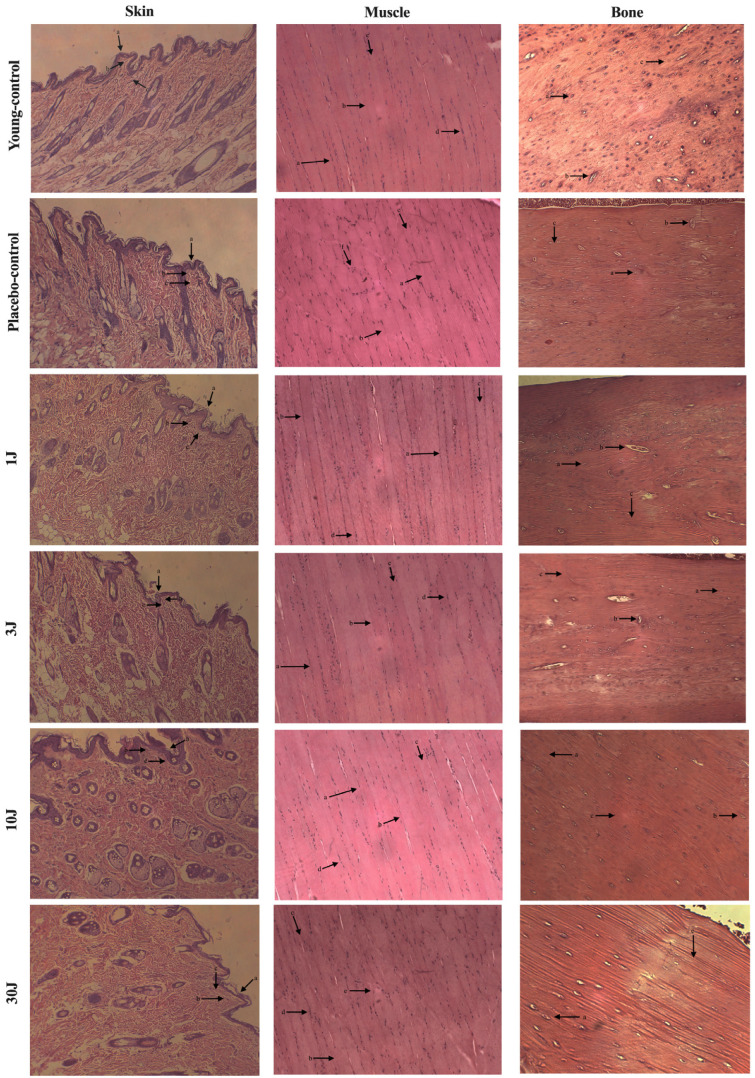
**Skin**: 1 J: (a) atrophy and decreased number of keratinocytes; (b) marked atrophy; (c) thin and short collagen fibers. 3 J: (a) atrophy and decreased number of keratinocytes; (b) marked atrophy; (c) thin and short collagen fibers. 10 J: (a) atrophy and decreased number of keratinocytes; (b) marked atrophy; (c) thin and short collagen fibers. 30 J: (a) atrophy and decreased number of keratinocytes; (b) marked atrophy; (c) thin and short collagen fibers. Placebo-control: (a) atrophy and decreased number of keratinocytes; (b) marked atrophy; (c) thin and short collagen fibers. Young-control: (a) atrophy and decreased number of keratinocytes; (b) marked atrophy; (c) thin and short collagen fibers. **Muscle**: 1 J: (a) organized muscle fibers; (b) large fiber diameter; (c) elongated fibers; (d) multinucleated fibers, with nuclei at the periphery. 3 J: (a) organized muscle fibers; (b) large fiber diameter; (c) elongated fibers; (d) multinucleated fibers, with nuclei at the periphery. 10 J: (a) organized muscle fibers; (b) large fiber diameter; (c) elongated fibers; (d) multinucleated fibers, with nuclei at the periphery. 30 J: (b) large fiber diameter; (c) elongated fibers; (d) multinucleated fibers, with nuclei at the periphery; (e) disorganized muscle fibers. Placebo-control: (a) organized muscle fibers; (b) large fiber diameter; (c) elongated fibers; (f) nuclei dislocated to center. Young-control: (a) organized muscle fibers; (b) large fiber diameter; (c) elongated fibers; (d) multinucleated fibers, with nuclei at the periphery. **Bone** (**cortical zone**): 1 J: (a) presence of osteocytes; (b) presence of Haversian Canal; (c) preserved bone tissue. 3 J: (a) presence of osteocytes; (b) presence of Haversian Canal; (c) preserved bone tissue. 10 J: (a) presence of osteocytes; (b) presence of Haversian Canal; (c) preserved bone tissue. 30 J: (a) presence of osteocytes; (c) preserved bone tissue. Placebo-control: (a) presence of osteocytes; (b) presence of Haversian Canal; (c) preserved bone tissue. Young-control: (a) presence of osteocytes; (b) presence of Haversian Canal; (c) preserved bone tissue.

Figure 1 shows the morphological aspects of the skin, muscle, and bone at the irradiation site for 15 weeks. With respect to the skin, all the groups presented with normal signs of aging in the epithelial tissue, papillary dermis, and reticular dermis. In addition, no difference between the groups in muscle morphology regarding organization, diameter, length, and nuclei of the fibers was observed. A slight change in fiber organization in the 30 J group and the fiber nuclei in the placebo control group was noted as an exception. Lastly, with respect to the bone, all the structures in the cortical zone were preserved in all the groups. The absence of the Haversian Canal in the 30 J group was noted as an exception.

#### 3.1.2. Medium-Term Treatment (30 Weeks)

**Figure 2:**
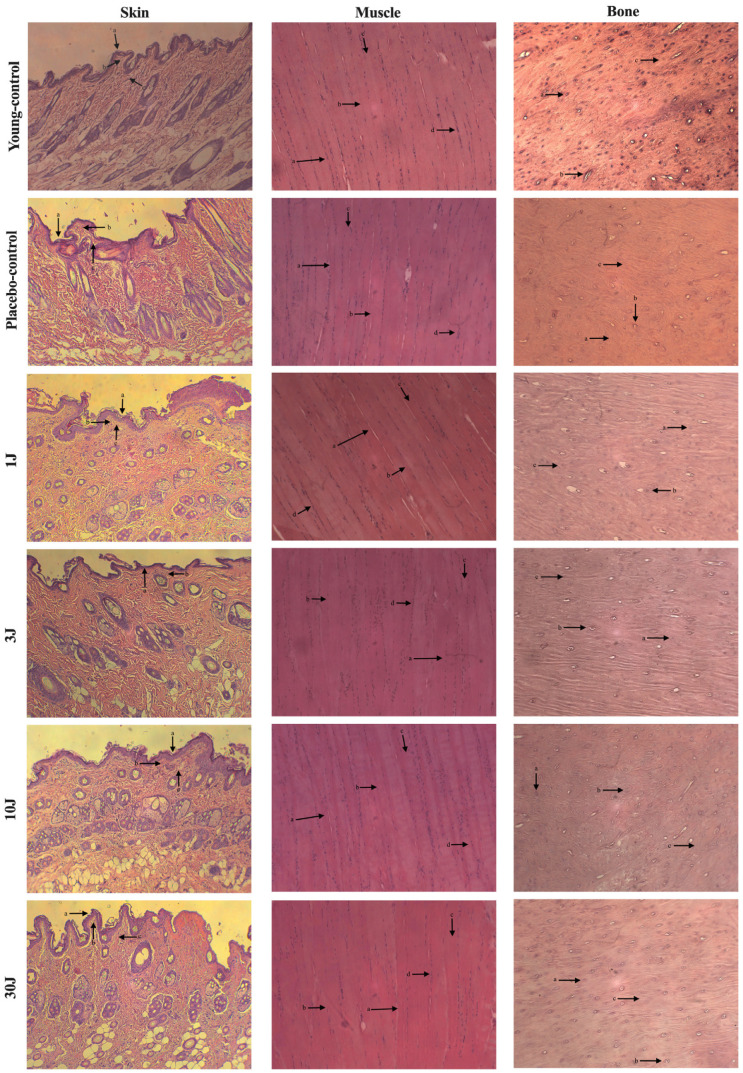
**Skin**: 1 J: (a) atrophy and decreased number of keratinocytes; (b) marked atrophy; (c) thin and short collagen fibers. 3 J: (a) atrophy and decreased number of keratinocytes; (b) marked atrophy. 10 J: (a) atrophy and decreased number of keratinocytes; (b) marked atrophy; (c) thin and short collagen fibers. 30 J: (a) atrophy and decreased number of keratinocytes; (b) marked atrophy; (c) thin and short collagen fibers. Placebo-control: (a) atrophy and decreased number of keratinocytes; (b) marked atrophy; (c) thin and short collagen fibers. Young-control: (a) atrophy and decreased number of keratinocytes; (b) marked atrophy; (c) thin and short collagen fibers. **Muscle**: 1J: (a) organized muscle fibers; (b) large fibers diameter; (c) elongated fibers; (d) multinucleated fibers, with nuclei at the periphery. 3 J: (a) organized muscle fibers; (b) large fiber diameter; (c) elongated fibers; (d) multinucleated fibers, with nuclei at the periphery. 10 J: (a) organized muscle fibers; (b) large fiber diameter; (c) elongated fibers; (d) multinucleated fibers, with nuclei at the periphery. 30 J: (a) organized muscle fibers; (b) large fiber diameter; (c) elongated fibers; (d) multinucleated fibers, with nuclei at the periphery. Placebo-control: (a) organized muscle fibers; (b) large fiber diameter; (c) elongated fibers; (d) multinucleated fibers, with nuclei at the periphery. Young-control: (a) organized muscle fibers; (b) large fiber diameter; (c) elongated fibers; (d) multinucleated fibers, with nuclei at the periphery. **Bone** (**cortical zone**): 1 J: (a) presence of osteocytes; (b) presence of Haversian Canal; (c) preserved bone tissue. 3 J: (a) presence of osteocytes; (b) presence of Haversian Canal; (c) preserved bone tissue. 10 J: (a) presence of osteocytes; (b) presence of Haversian Canal; (c) preserved bone tissue. 30 J: (a) presence of osteocytes; (b) presence of Haversian Canal; (c) preserved bone tissue. Placebo-control: (a) presence of osteocytes; (b) presence of Haversian Canal; (c) preserved bone tissue. Young-control: (a) presence of osteocytes; (b) presence of Haversian Canal; (c) preserved bone tissue.

Figure 2 shows the morphological aspects of the skin, muscle, and bone at the irradiation site for 30 weeks. All the groups presented with normal signs of aging in the epithelial tissue, papillary dermis, and reticular dermis. In addition, no difference between the groups in muscle morphology regarding organization, diameter, length, and nuclei of the fibers was observed. Finally, with respect to the bone, all the structures in the cortical zone were preserved in all the groups.

#### 3.1.3. Long-Term Treatment (60 Weeks)

**Figure 3:**
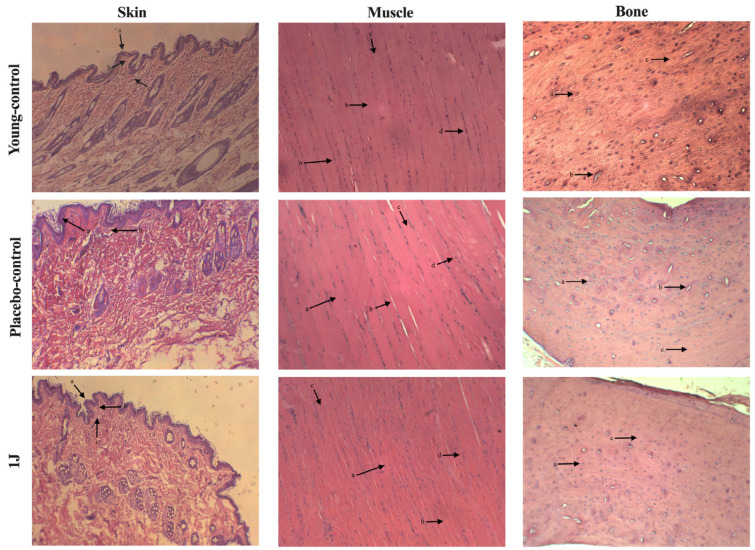

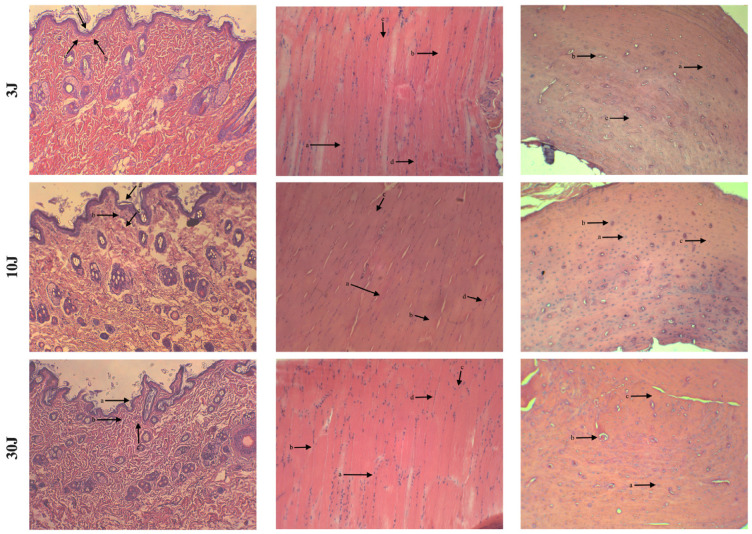
**Skin**: 1 J: (a) atrophy and decreased number of keratinocytes; (b) marked atrophy; (c) thin and short collagen fibers. 3 J: (a) atrophy and decreased number of keratinocytes; (b) marked atrophy; (c) thin and short collagen fibers. 10 J: (a) atrophy and decreased number of keratinocytes; (b) marked atrophy; (c) thin and short collagen fibers. 30 J: (a) atrophy and decreased number of keratinocytes; (b) marked atrophy; (c) thin and short collagen fibers. Placebo-control: (a) atrophy and decreased number of keratinocytes; (b) marked atrophy. Young-control: (a) atrophy and decreased number of keratinocytes; (b) marked atrophy; (c) thin and short collagen fibers. **Muscle**: 1 J: (a) organized muscle fibers; (b) large fiber diameter; (c) elongated fibers; (d) multinucleated fibers, with nuclei at the periphery. 3 J: (a) organized muscle fibers; (b) large fiber diameter; (c) elongated fibers; (d) multinucleated fibers, with nuclei at the periphery. 10 J: (a) organized muscle fibers; (b) large fiber diameter; (c) elongated fibers; (d) multinucleated fibers, with nuclei at the periphery. 30 J: (a) organized muscle fibers; (b) large fiber diameter; (c) elongated fibers; (d) multinucleated fibers, with nuclei at the periphery. Placebo-control: (a) organized muscle fibers; (b) large fiber diameter; (c) elongated fibers; (d) multinucleated fibers, with nuclei at the periphery. Young-control: (a) organized muscle fibers; (b) large fiber diameter; (c) elongated fibers; (d) multinucleated fibers, with nuclei at the periphery. **Bone** (**cortical zone**): 1 J: (a) presence of osteocytes; (c) preserved bone tissue. 3 J: (a) presence of osteocytes; (b) presence of Haversian Canal; (c) preserved bone tissue. 10 J: (a) presence of osteocytes; (b) presence of Haversian Canal; (c) preserved bone tissue. 30 J: (a) presence of osteocytes; (b) presence of Haversian Canal; (c) preserved bone tissue. Placebo-control: (a) presence of osteocytes; (b) presence of Haversian Canal; (c) preserved bone tissue. Young-control: (a) presence of osteocytes; (b) presence of Haversian Canal; (c) preserved bone tissue.

Figure 3 shows the morphological features of skin, muscle, and bone at the irradiation site for 60 weeks. With respect to the skin, all groups presented with normal signs of aging in the epithelial tissue, papillary dermis, and reticular dermis. In addition, no difference between the groups in muscle morphology regarding organization, diameter, length, and nuclei of the fibers was observed. Lastly, with respect to the bone, all the structures in the cortical zone were preserved in all the groups. The absence of the Haversian Canal in the 1 J group was considered an exception.

### 3.2. Biochemical Analysis: GPT, GOT, Urea, Creatinine, and VEGF

#### 3.2.1. Short-Term Treatment (15 Weeks)

**Figure 4:**
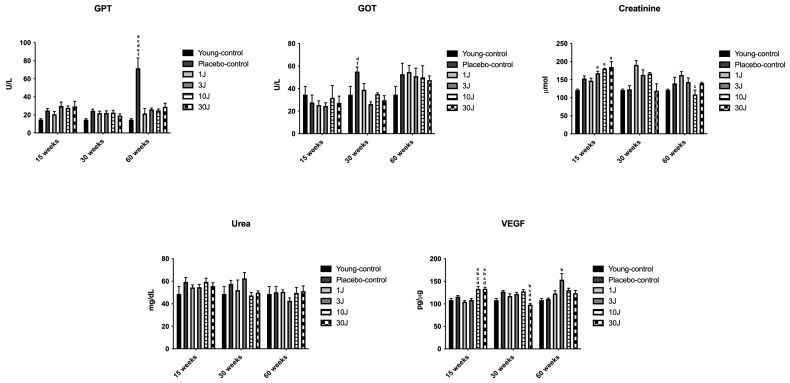
Data graph of the mean and standard error of the mean of the biochemical analysis. ^a^ young-control, ^b^ placebo-control, ^c^ 1 J, ^d^ 3 J, ^e^ 10 J, and ^f^ 30 J.

No differences between the groups were observed at baseline (*p* > 0.05) in the GPT, GOT, urea, creatinine, and VEGF levels. Moreover, no differences between the groups were observed at 15 weeks in the GPT, GOT, and urea levels. In contrast, we observed an increase in creatinine levels in the 3 J (*p* = 0.0273), 10 J (*p* = 0.0032), and 30 J (*p* = 0.0022) groups compared to those in the young control group. In addition, there was an increase in VEGF expression in the 10 J and 30 J groups compared to that in the young control (*p* = 0.0079; *p* = 0.0088), placebo-control (*p* = 0.0076; *p* = 0.0109), 1 J (*p* < 0.0001; *p* < 0.0001), and 3 J (*p* < 0.0001; *p* = 0.0002) groups. Table 2 and Figure 4 summarize the results of the biochemical analyses.

#### 3.2.2. Medium-Term Treatment (30 Weeks)

No differences between the groups were observed at baseline (*p* > 0.05) in the GPT, GOT, urea, creatinine, and VEGF levels. Moreover, no differences between the groups were observed at 30 weeks in the creatinine and urea levels. In contrast, there was an increase in GPT levels in the placebo control group compared to those in the young control group (*p* = 0.0216). In addition, there was an increase in GOT levels in the placebo control group compared to those in the 3 J (*p* = 0.0027) and 30 J (*p* = 0.0067) groups. Lastly, there was a decrease in VEGF in the 30 J group compared to that in the placebo control (*p* < 0.0001), 1 J (*p* = 0.0015), 3 J (*p* < 0.0001), and 10 J (*p* < 0.0001) groups. Table 2 and Figure 4 summarize the results of the biochemical analyses.

#### 3.2.3. Long-Term Treatment (60 Weeks)

No differences between the groups were observed at baseline (*p* > 0.05) in the GPT, GOT, urea, creatinine, and VEGF levels. Moreover, no differences between the groups were observed at 60 weeks in the GOT or urea levels. In contrast, there was an increase in GPT levels in the placebo group compared to those in all the experimental groups (*p* < 0.0001). In addition, we observed a decrease in creatinine levels in the 10 J group compared to those in the 1 J group (*p* = 0.0462). Lastly, there was an increase in VEGF in the 3 J group when compared to that in the placebo-control group (*p* = 0.0133). Table 2 and Figure 4 summarize the results of the biochemical analyses.

## 4. Discussion

This study investigated the detrimental and protective effects of short-term (15 weeks), medium-term (30 weeks), and long-term treatment (60 weeks) with doses of 1, 3, 10, and 30 J PBMT-sMF during the aging process in rats. Prolonged treatment with PBMT-sMF did not have detrimental effects on the skin, muscle, bone, liver, or kidneys. However, some protective effects were observed; PBMT-sMF decreased GPT levels during long-term treatment and increased VEGF levels during short-term treatment with higher doses and during long-term treatment with lower doses. Finally, PBMT-sMF 30 J decreased VEGF levels following medium-term treatment.

Aging is a systemic process defined as the accumulation of deleterious changes with advancing age in cells and different tissues such as the skin, muscle, bone, liver, and kidney [35]. With respect to the skin, our results demonstrated clear signs of aging in the animals from all groups treated with the placebo or PBMT-sMF, regardless of dose, compared to the animals in the young control group. A decreased number of epithelial cells, marked atrophy, and a reduced number of collagen fibers were observed. These findings corroborate previous studies that evidenced a decrease in the rate of epidermal regeneration, barrier function, and elasticity due to a reduction in the amount of collagen and degeneration of elastic fibers [33,36]. Moreover, this decrease occurred in addition to the decrease in skin thickness due to atrophy and disorientation of the epidermis, dermis, and subcutaneous fat with aging [37]. A previous study evaluated the effects of PBMT on cutaneous wound healing in aged rats and observed its effectiveness in modulating inflammatory mediators and increasing collagen production. However, unlike our current study, this previous animal study [19] evaluated non-intact skin, that is, injured skin. In addition, different outcomes were evaluated, except for the VEGF levels. Lastly, PBMT was applied acutely.

In skeletal muscle tissue, there is a decrease in the size and number of muscle fibers with aging in addition to muscle capillarization and an increase in muscle cross-section [38,39]. Evidence suggests that PBMT decreases muscle damage after injury by organizing the muscle fibers and cell nuclei [26]. Additionally, PBMT increases muscle fiber diameter and cross-sectional muscle repair following peripheral nerve injury [40]. In contrast, in this study, we observed little or no change in the muscle tissue between the groups. However, we evaluated intact tissue, whereas, in the aforementioned studies, injured tissue was studied.

Bone loss is observed in the bone tissue during aging. A previous study demonstrated that PBMT improved tibial bone repair in osteoporotic rats after an induced bone defect by increasing the deposition and improving the organization of collagen fibers [41]. Moreover, PBMT enhances fracture repair by increasing bone mineral density and callus volume [42,43]. In contrast, we observed only a slight difference between the groups, demonstrating that PBMT did not promote prophylactic or detrimental effects on the bone tissue. However, we evaluated intact tissues with only signs of aging.

Aging also causes morphological and physiological changes in organs, such as the liver, promoting a reduction in blood flow and volume in addition to a lower immune response. These changes can affect the metabolism of substances and increase susceptibility to liver injury [44]. Our results demonstrated that aging promoted hepatic changes in the placebo-control group as measured by the GPT levels after 30 and 60 weeks of treatment and GOT levels after 30 weeks of treatment. In contrast, we observed that PBMT-sMF at all doses protected the liver from the deleterious effects of aging and decreased the GPT levels after 60 weeks and GOT levels after 30 weeks of treatment. Previous studies have demonstrated that PBMT reduces acute hepatic oxidative stress in a diabetic rat model, improves liver injury after ischemia–reperfusion injury, and improves liver regeneration after hepatectomy [30,31,45]. Contrary to our study, in the above-mentioned studies, a common aspect was that the animals had liver injury before treatment with PBMT. However, we observed that these positive effects on the liver could also be promoted in intact tissue. In addition, GPT and GOT levels were analyzed as indicators of toxicity in the PBMT-sMF safety evaluation and pharmaceutical studies. Furthermore, we realized that treatment with PBMT-sMF at all doses was safe, as it did not promote hepatotoxicity, even after a prolonged period of irradiation (from 15 to 60 weeks).

Creatinine and urea are the biochemical markers commonly used to evaluate renal function. In our study, creatinine levels increased in the PBMT-sMF 3 J, 10 J, and 30 J groups after 15 weeks of treatment. However, this increase was observed only in the younger animals in the young-control group. In addition, no difference in urea levels between the groups was observed. Contrary to our results, a previous study evaluated the effects of acute irradiation with PBMT on ischemia–reperfusion injury in the kidneys of diabetic rats and observed a decrease in creatinine levels in the animals treated with PBMT [46]. Another study investigated the effects of PBMT irradiation on stem cells in the bone marrow of rats after acute ischemia–reperfusion injury. This study also found that animals treated with PBMT had lower levels of creatinine and blood urea [47]. The abovementioned studies demonstrated some protective effects of PBMT on injured kidneys; however, our study demonstrated that these effects were not observed in intact tissue. In contrast, we found that PBMT-sMF did not have detrimental effects on the kidneys, even after prolonged therapy (15 to 60 weeks).

We observed that PBMT-sMF at higher doses of 10 and 30 J promoted an increase in VEGF following short-term treatment, whereas the lower dose of 3 J promoted an increase after long-term treatment. Corroborating our results, a previous study demonstrated that PBMT at a low dose of 2 J was able to increase VEGF in skin wound repair induced in aged rats [19]. Another study demonstrated that low doses of PBMT using infrared and red low-level lasers increased VEGF levels in diabetic muscle-injured rats [48]. However, unlike our study, the abovementioned studies evaluated only one dose (a low dose) of PBMT; the treatment was very short (maximum 14 days), and the evaluated tissue was injured. Therefore, although the characteristics of these studies, including ours, were diverse, their results were similar.

Over the years, studies have demonstrated the effects of PBMT on injured tissues, either by the injury itself or secondarily by some conditions, such as diabetes. In contrast, no studies have evaluated the effects of prolonged use (15–60 weeks, for instance) of PBMT on intact tissues, only presenting changes as a result of aging. This makes a head-to-head comparison of their evidence with our results difficult.

PBMT can promote prophylactic effects in healthy and intact tissues due to its increased cellular metabolism [49]. However, our results demonstrated that PBMT-sMF at all the tested doses did not reduce the normal and expected signs of aging of the skin, muscle, and bone, as observed through morphological analysis. The results also demonstrated that there were no detrimental effects, even after a prolonged treatment period (15–60 weeks). Similar results were observed for the urea levels, which did not differ between the groups. In contrast, we observed an increase in creatinine in the groups treated with PBMT-sMF after 15 weeks compared to the young control group. However, there was no difference compared to the placebo-control group, suggesting that this increase in creatine was inherent to aging and not to the treatment used. Therefore, our results suggest that PBMT-sMF does not promote detrimental effects in toxic signaling organs, such as the liver and kidney. In contrast, our results demonstrated that PBMT-sMF, at all the doses tested, protected the liver against the inherent deleterious effects of aging. Furthermore, the increase in VEGF after treatment with PBMT-sMF 3 J could also indicate a possible prophylactic effect of preserving muscle tissue during aging, as VEGF plays a fundamental role in angiogenesis, induces vasodilation, increases vascular permeability, and stimulates the proliferation, migration, and survival of endothelial cells [50]. In regard to VEGF, we observed that PBMT-sMF 30 J decreased the VEGF levels after 30 weeks of treatment; however, these values normalized after 60 weeks of treatment. After analyzing the data, we did not find a plausible explanation for this decrease, which requires further investigation in future studies. In summary, our results demonstrated that PBMT-sMF promoted prophylactic effects during the aging process and did not cause any deleterious effects in these tissues. Therefore, treatment with PBMT-sMF may be safe, even after a long period (15–60 weeks).

One of the strengths of this study was the large number of treatments (once a day, three times a week for 15, 30, and 60 consecutive weeks). In addition, the analyses were performed over very long time points (up to 60 weeks). In contrast, the limitations were that we tested only four doses; we did not analyze organs such as the brain, lungs, and heart, and we did not perform gene expression analyses of different tissues and markers.

Further studies are required to better investigate the effects of PBMT-sMF on VEGF, as we found that 30 J PBMT-sMF decreased VEGF levels after 30 weeks of treatment, but these values normalized after 60 weeks of treatment.

## 5. Conclusions

PBMT-sMF did not have any detrimental effects on the skin, muscle, bone, kidney, or liver after short-, medium-, and long-term treatments in aging rats. These findings may translate into PBMT-sMF without side effects and/or adverse events. In addition, PBMT-sMF may have protective effects on the muscle tissue after short- and long-term treatment in aging rats. For short-term treatments a dose of 10 J, delivered over 77 s per site with direct contact to the skin, three times a week should be used. For long-term treatments, a dose of 3 J delivered over 23 s per site with direct contact to the skin, three times a week showed prophylactic effects in the target tissue without adverse/detrimental effects.

## Figures and Tables

**Table 1:** PBMT-sMF parameters.

Parameter (Unit)	Value or Method
Number of lasers	1 Super-pulsed infrared
Wavelength (nm)	905 (±1)
Frequency (Hz)	250
Peak power (W)	25
Average mean optical output (mW)	0.625
Power density (mW/cm^2^)	1.42
Energy density (J/cm^2^)	0.011, 0.033, 0.11, or 0.33
Dose (J)	0.005, 0.0144, 0.048, or 0.144
Spot size of laser (cm^2^)	0.44
Number of red LEDs	4 Red
Wavelength of red LEDs (nm)	640 (±10)
Frequency (Hz)	2
Average optical output (mW)—each	15
Power density (mW/cm^2^)—each	16.67
Energy density (J/cm^2^)—each	0.133, 0.383, 1.283, or 3.84
Dose (J)—each	0.12, 0.345, 1.155, or 3.46
Spot size of red LED (cm^2^)—each	0.9
Number of infrared LEDs	4 Infrared
Wavelength of infrared LEDs (nm)	875 (±10)
Frequency (Hz)	16
Average optical output (mW)—each	17.5
Power density (mW/cm^2^)—each	19.44
Energy density (J/cm^2^)—each	0.155, 0.447, 1.497, or 4.490
Dose (J)—each	0.14, 0.4025, 1.3475, or 4.042
Spot size of infrared LED (cm^2^)—each	0.9
Magnetic field (mT)	35
Irradiation time per site (s)	8, 23, 77, or 231
Total dose per site (J)	1.0, 3.0, 10.0, or 30.0
Total dose applied in muscular group (J)	1.0, 3.0, 10.0, or 30.0
Aperture of device (cm^2^)	0.394
Device total power output (mW)	130.62
Device power density (mW/cm^2^)	331.54
Device energy density (J/cm^2^)	2.54, 7.61, 25.38, or 76.14
Application mode	Cluster probe held stationary in contact with the skin at a 90° angle while applying slight pressure

**Table 2:** Mean and standard deviation of the biochemical analysis.

Outcomes	Mean (Standard Deviation)					
	Young-Control	Placebo-Control	1 J	3 J	10 J	30 J
**Glutamic pyruvic transaminase** (**GPT**) (**U/L**)						
15 weeks	14.71 (2.58)	24.91 (5.22)	20.70 (6.85)	29.84 (9.81)	27.67 (5.12)	29.34 (12.84)
30 weeks	14.71 (2.58)	24.56 (4.09) ^a^	22.04 (4.88)	22.23 (5.02)	22.46 (5.77)	19.13 (4.39)
60 weeks	14.71 (2.58)	71.70 (25.60) ^a,c,d,e,f^	21.52 (12.46)	26.03 (3.64)	24.97 (3.61)	28.95 (8.94)
**Glutamic oxaloacetic transaminase** (**GOT**) (**U/L**)						
15 weeks	34.64 (16.43)	27.61 (14.90)	25.39 (8.42)	24.61 (6.46)	31.74 (24.53)	27.22 (13.68)
30 weeks	34.64 (16.43)	55.10 (9.01) ^d,f^	38.95 (12.36)	26.32 (4.28)	35.10 (2.48)	29.66 (9.23)
60 weeks	34.64 (16.43)	52.69 (22.01)	54.66 (13.25)	51.02 (16.15)	49.97 (23.17)	47.47 (8.57)
**Creatinine** (**μmol**)						
15 weeks	121.20 (6.09)	153.30 (15.91)	147.20 (15.42)	167.80 (12.50) ^a^	180.00 (4.11) ^a^	184.80 (34.98) ^a^
30 weeks	121.20 (6.09)	123.40 (22.56)	191.00 (27.02)	163.10 (31.50)	167.00 (6.13)	119.20 (43.79)
60 weeks	121.20 (6.09)	139.70 (38.23)	162.90 (20.56)	143.50 (25.27)	109.10 (27.43) ^c^	140.60 (5.23)
**Urea** (**mg/dL**)						
15 weeks	48.75 (14.82)	59.42 (8.88)	54.48 (5.12)	54.72 (5.77)	59.45 (7.42)	55.74 (6.56)
30 weeks	48.75 (14.82)	57.54 (7.15)	52.12 (20.14)	62.47 (11.79)	47.36 (6.30)	49.87 (3.49)
60 weeks	48.75 (14.82)	50.17 (11.95)	50.56 (4.17)	42.72 (5.61)	49.70 (10.80)	51.16 (10.53)
**Vascular endothelial growth factor** (**VEGF**) (**pg/μg**)						
15 weeks	108.30 (8.06)	116.10 (5.44)	104.80 (5.09)	108.60 (7.94)	133.00 (12.18) ^a,b,c,d^	133.60 (7.40) ^a,b,c,d^
30 weeks	108.30 (8.06)	127.10 (5.08)	117.40 (11.16)	121.90 (7.91)	127.70 (8.34)	98.26 (5.09) ^b,c,d,e^
60 weeks	108.30 (8.06)	110.70 (4.80)	122.90 (14.37)	153.60 (30.50) ^b^	130.20 (10.25)	123.60 (13.77)

^a^ young-control, ^b^ placebo-control, ^c^ 1 J, ^d^ 3 J, ^e^ 10 J, and ^f^ 30 J.

## Data Availability

The datasets generated and analyzed during the current study are available from the corresponding author upon reasonable request.
